# Psychosocial stress at work and perceived quality of care among clinicians in surgery

**DOI:** 10.1186/1472-6963-11-109

**Published:** 2011-05-20

**Authors:** Jens Klein, Kirstin Grosse Frie, Karl Blum, Olaf von dem Knesebeck

**Affiliations:** 1 University Medical Center Hamburg-Eppendorf, Center for Psychosocial Medicine, Department of Medical Sociology and Health Economics, Martinistr. 52, 20246 Hamburg, Germany; 2 Deutsches Krankenhausinstitut, Hansaallee 210, 40549 Duesseldorf, Germany

## Abstract

**Background:**

Little is known about the association between job stress and job performance among surgeons, although physicians' well-being could be regarded as an important quality indicator. This paper examines associations between psychosocial job stress and perceived health care quality among German clinicians in surgery.

**Methods:**

Survey data of 1,311 surgeons from 489 hospitals were analysed. Psychosocial stress at work was measured by the effort-reward imbalance model (ERI) and the demand-control model (job strain). The quality of health care was evaluated by physicians' self-assessed performance, service quality and error frequency. Data were collected in a nationwide standardised mail survey. 53% of the contacted hospitals sent back the questionnaire; the response rate of the clinicians in the participating hospitals was about 65%. To estimate the association between job stress and quality of care multiple logistic regression analyses were conducted.

**Results:**

Clinicians exposed to job stress have an increased risk of reporting suboptimal quality of care. Magnitude of the association varies depending on the respective job stress model and the indicator of health care quality used. Odds ratios, adjusted for gender, occupational position and job experience vary between 1.04 (CI 0.70-1.57) and 3.21 (CI 2.23-4.61).

**Conclusion:**

Findings indicate that theoretical models of psychosocial stress at work can enrich the analysis of effects of working conditions on health care quality. Moreover, results suggest interventions for job related health promotion measures to improve the clinicians' working conditions, their quality of care and their patients' health.

## Background

Job stress and decreased well-being are highly prevalent among physicians [1,2] and especially among surgeons [3,4]. Studies have examined a range of contributing factors to physicians' distress: schedule, workload, overtime expectations, sleep deprivation, relationships with co-workers, delayed gratifications, limited control and a loss of autonomy, work-life imbalance, feelings of isolation and a lack of time for research activities [1,3,5-7]. Reduced mental and physical health [8], depression and symptoms of burnout like emotional exhaustion, depersonalization or lower personal accomplishment were frequently identified as harmful consequences for physicians [1-3,9-11]. The associations between physicians' and especially surgeons' stress and their job performance are analysed to a lesser extent. So, physicians' well-being could be regarded as an important but missing quality indicator [2]. Therefore, this study analyses the associations between psychosocial stress at work and the quality of health care among clinicians in surgery.

For the estimation of job stress, two established work stress models were used: the demand-control model (job strain) [12] and the effort-reward imbalance model (ERI) [13]. The demand-control model postulates that job strain results from the combination of high (quantitative) job demands and low job control that is subdivided into skill discretion and decision authority. The ERI-model is focused on the experienced lack of social reciprocity. An imbalance between high efforts and low rewards in terms of esteem, salary/job promotion or job security leads to negative emotions and harmful stress. Moreover, the model consists of an intrinsic component (overcommitment) which means a motivational pattern of excessive work-related commitment and a high need for approval. Overcommitment can amplify an effort-reward imbalance or can cause emotional stress independently. Both models assume that job stress leads to a reduced physical and/or mental health. This assumption was confirmed in numerous studies [14-17] some of them among physicians [6-8,18]. While some studies have analysed associations between burnout and/or depression and quality of care among physicians [19-21] and surgeons [9,22], relations between job stress and quality of health care were rarely examined [23-25]. Furthermore, these studies differ considerably in their design, sample and measurement of stress. Indeed, most of these studies indicate a higher risk of medical errors and suboptimal patient care among stressed and/or burnt out and depressed physicians [9,10,19-24]. Moreover, the majority of the investigations were not based on a theoretical model of job stress. Only one study used the ERI- and the demand-control model [26] to explore the association between job stress and quality of care and found that infection rates of the ward correlated with high effort-reward imbalance among the staff but not with job strain. Against this background, we examine the association between two job stress models (ERI and job strain) and various dimensions of perceived health care quality among German physicians in surgery.

## Methods

### Study Sample

The population consists of all clinicians in surgery working in general hospitals in Germany with a capacity of minimum 100 beds including a general surgical and/or gynaecological ward. The data collection was based on a stratified probability sample to represent large hospitals with more clinicians adequately. Between March and June 2008, general surgery and gynaecological wards of 922 hospitals were requested to take part in a standardised mail survey. In large hospitals (≥ 600 beds), 9 physicians were selected randomly whilst in smaller hospitals (100-599 beds) 3 physicians were chosen, resulting in a total of 3,648 potential respondents. Data of 489 hospitals and 1,311 clinicians were collected. Some sample characteristics are shown in Table 1.

**Table 1:** Sample characteristics (n = 1,311 clinicians in surgery), n (%) or mean [M] (standard deviations) [SD]

Age in years, M (SD)	39.1 (9.6)
**Gender**	
Male	789 (60.2)
Female	522 (39.8)

**Specialty**	
General surgery	967 (73.8)
Gynaecology/obstetrics	344 (26,2)

**Occupational position**	
Chief physicians	118 (9.0)
Senior physicians	273 (20.8)
Residents with advanced training	316 (24.1)
Residents without advanced training	604 (46.1)

**Job experience **(years), M (SD)	11.4 (9.6)

**Employment in clinic **(years), M (SD)	6.8 (7.5)

**Working hours per day**, M (SD)	9.9 (1.3)

### Measures

Psychosocial stress at work was measured by validated questionnaires based on the respective job stress model. The effort-reward imbalance (ERI) questionnaire [15] consists of 16 items on a 5-point Likert scale. 6 items refer to the effort scale (Cronbach's Alpha 0.75) and 10 items to the reward scale (Cronbach's Alpha 0.82). To describe the imbalance between effort and reward the effort-reward ratio was established [15]. A ratio of more than 1 indicates an effort-reward imbalance. The model's intrinsic component overcommitment, a mental coping strategy, was additionally assessed by 6 items (Cronbach's Alpha 0.76) on a 5-point Likert scale. As there is no fixed cutpoint for overcommitment, the high-risk group is determined by the upper tertile. The demand-control model was measured by 16 items taken from the job content questionnaire (JCQ) [27]. The intensity of demand (8 items) and control (3 items regarding decision authority/5 items regarding skill discretion) were measured on a 4-point Likert scale (Cronbach's Alpha 0.74 and 0.78). By dichotomising the two scales (median), 4 categories can be distinguished [12]: "no strain" (low demands/high control), "active job" (high demands/high control), "passive job" (low demands/low control) and "job strain" (high demand/low control).

Due to its complex nature, quality of health care was measured by considering different aspects of patient care. Perceived quality of care concerning the clinicians' performance was assessed by using a modified short version (13 items) of a German self-assessment instrument called the Chirurgisches Qualitätssiegel (CQS) [28,29] which was developed according to the Canadian Physician Achievement Review (PAR) [30,31] (see Appendix). Generally, the CQS evaluates different aspects of patient care, using a 5-point Likert scale ranging from "very good" to "bad". An explorative factor analysis reveals three dimensions/subscales: psychosocial care (5 items, Cronbach's Alpha 0.81), diagnosis/therapy (4 items, Cronbach's Alpha 0.82) and quality assurance (4 items, Cronbach's Alpha 0.70). For the analyses, the subscales were dichotomised. The respective lower tertile was defined as suboptimal care. Additionally, the quality of patient care was measured by two questions about the frequency of diagnostic and therapeutic errors ("I have made mistakes in diagnosis"/"I have made mistakes in treatment") using a 4-point Likert scale ranging from "never" to "often". Both questions did not have a specific time of reference. A sum score was calculated and dichotomised with the upper tertile indicating a comparatively high error frequency. To assess service quality, the SERVQUAL [32,33], a multiple-item scale for measuring consumer perceptions of service quality, was used. SERVQUAL has been applied to explore service quality in hospitals and among health care staff in different studies [24,34,35]. In the present study, 12 items (with a 4-point Likert scale from Likert scale from "totally agree" to "totally disagree") were adopted to explore service quality on the surgical wards (see Appendix). Explorative factor analysis reveals two dimensions/subscales: organisation of care (4 items, Cronbach's Alpha 0.65) and patient orientation (8 items, Cronbach's Alpha 0.86). The subscales were dichotomised and the lower tertile of both dimensions indicates a low quality of health care.

### Statistical Analysis

To explore the association between job stress and perceived quality of care multivariate logistic regression analyses were applied, adjusted for gender, occupational position (subdivided into chief/senior physicians and residents with or without further education) and job experience (≤ 9 and > 9 years; median split). Odds ratios, 95% confidence intervals, Cox and Snell Pseudo-R^2^, Nagelkerke's Pseudo-R^2 ^and the significance level of the overall model are displayed. Statistical software PASW 18.0 was used. For the sake of completeness, odds ratios and confidence intervals of all control variables are additionally shown in the tables. However, they are not discussed in detail as the study is focused on the association between job stress and patient care.

## Results

The response rate was about 53% on hospital level, 36% on physician level and 65% regarding the clinicians in participating hospitals. Descriptive statistics concerning indicators of job stress and perceived quality of health care among German clinicians in surgery are displayed in Table 2. It shows that a quarter of the sample suffers from effort-reward imbalance and about 22% from job strain.

**Table 2:** Indicators of job stress and self-reported quality of health care among clinicians in surgery in Germany: Frequencies^a^, n (%) and means [M] (standard deviations) [SD]

	n (%) or M (SD)
**Effort-reward imbalance model**	

ERI > 1	301 (25.1)^b^
ERI	0.87 (0.37)^c^
Overcommitment (sum scale from 6 to 24)	15.7 (3.7)^c^

**Demand-control model**	

No strain (low demands/high control)	347 (26.9)^b^
Active job (high demands/high control)	256 (19.8)^b^
Passive job (low demands/low control)	399 (30.9)^b^
Job strain (high demands/low control)	289 (22.4)^b^

**Self-reported quality of health care**	

Dimensions according to the CQS/PAR^d^	
Psychosocial care (sum scale from 5 to 25)	19.7 (3.1)^c^
Diagnosis/therapy (sum scale from 4 to 20)	15.9 (2.1)^c^
Quality assurance (sum scale from 4 to 20)	12.6 (2.6)^c^

Dimensions according to the SERVQUAL	
Organisation of care (sum scale from 4 to 16)	11.0 (1.7)^c^
Patient orientation (sum scale from 8 to 32)	24.4 (3.4)^c^

Medical errors (sum scale from 2 to 8) ^e^	4.22 (0.87)^c^

^a^Variances in number of cases due to missing data

^b^n (%)

^c^M (SD)

^d^Chirurgisches Qualitätssiegel/Physician Achievement Review

^e^A higher score indicates a higher error frequency

The associations between both job stress models and the indicators of perceived quality of health care are displayed in Tables 3 and 4 by means of odds ratios and confidence intervals. In Table 3 the three dimensions of the CQS (psychosocial care, diagnosis/therapy and quality assurance) are introduced as dependent variables. There is a significant association between ERI and psychosocial care according to the CQS. Furthermore, suboptimal care according to the CQS is significantly associated with job strain after the demand-control model with odds ratios varying between 1.6 and 2.6 in the different quality dimensions. Additionally, a passive job according to the demand-control model is associated with a lower quality of care concerning all indicators of the CQS. As shown in Table 4, the two subscales of the SERVQUAL (organisation of care and patient orientation) are significantly associated with effort-reward imbalance as well as with job strain (odds ratios vary between 1.9 and 3.2). Surgeons describing their job as active (high demands/high control) or passive (low demands/low control) also more frequently report suboptimal care in terms of organisation and patient orientation. Associations between psychosocial stress at work (both models) and frequency of self-reported medical errors are not significant except for overcommitment.

**Table 3:** Effort-reward imbalance, job strain and self-rated quality of care (CQS/PAR^a^; lower tertile) controlling for gender, occupational position, job experience: Odds ratios^b ^(OR) and 95% confidence intervals (CI)

	Psychosocial care	**Diagnosis/therapy**	Quality assurance
	
	OR (95% CI)	OR (95% CI)	OR (95% CI)
**Effort-reward imbalance model**			
ERI ≤ 1	1	1	1
ERI > 1	**1.38 (1.03-1.86)**	1.12 (0.82-1.54)	1.15 (0.85-1.56)
Overcommitment (lower two tertiles)	1	1	1
Overcommitment (upper tertile)	0.78 (0.59-1.03)	1.28 (0.96-1.72)	0.96 (0.73-1.28)
**Control variables**			
Male	1	1	1
Female	**0,75 (0.58-0,98)**	**1.74 (1.33-2.28)**	0.96 (0.73-1.25)
Chief/senior physicians	1	1	1
Residents	1.18 (0.81-1.72)	**4.10 (2.58-6.51)**	**1.60 (1.08-2.36)**
Job experience > 9 years	1	1	1
Job experience ≤ 9 years	**1.71 (1.23-2.37)**	**2.60 (1.87-3.62)**	**2.16 (1.55-3.00)**
Cox and Snell Pseudo-R^2^	0.029	0.178	0.059
Nagelkerke's Pseudo-R^2^	0.040	0.246	0.082
Significance level (Chi^2^)	0.000	0.000	0.000

**Demand-control model**			
No strain (low demands/high control)	1	1	1
Active job (high demands/high control)	**1.88 (1.29-2.74)**	1.39 (0.90-2.15)	1.06 (0.72-1.57)
Passive job (low demands/low control)	**2.01 (1.40-2.87)**	**3.28 (2.24-4.81)**	**1.43 (1.01-2.03)**
Job strain (high demands/low control)	**2.58 (1.77-3.75)**	**2.09 (1.40-3.13)**	**1.64 (1.13-2.37)**
**Control variables**			
Male	1	1	1
Female	**0.71 (0.55-0.92)**	**1.63 (1.25-2.12)**	0.92 (0.71-1.19)
Chief/senior physicians	1	1	1
Residents	1.11 (0.76-1.61)	**3.13 (1.98-4.96)**	1.46 (0.99-2.16)
Job experience > 9 years	1	1	1
Job experience ≤ 9 years	**1.55 (1.13-2.13)**	**2.85 (2.06-3.93)**	**2.15 (1.56-2.96)**
Cox and Snell Pseudo-R^2^	0.046	0.214	0.068
Nagelkerke's Pseudo-R^2^	0.064	0.296	0.095
Significance level (Chi^2^)	0.000	0.000	0.000

^a^Chirurgisches Qualitätssiegel/Physician Achievement Review

^b^Significant odds ratios are bold

**Table 4:** Effort-reward imbalance, job strain and self-rated quality of care (SERVQUAL; lower tertile), medical errors (upper tertile) controlling for gender, occupational position, job experience: Odds ratios^a ^(OR) and 95% confidence intervals (CI)

	Organisation of care	Patient orientation	Medical errors
	
	OR (95% CI)	OR (95% CI)	OR (95% CI)
**Effort-reward imbalance model**			
ERI ≤ 1	1	1	1
ERI > 1	**2.33 (1.76-3.09)**	**1.90 (1.43-2.53)**	1.24 (0.90-1.69)
Overcommitment (lower two tertiles)	1	1	1
Overcommitment (upper tertile)	1.29 (0.99-1.67)	0.94 (0.73-1.23)	**1.36 (1.01-1.82)**
**Control variables**			
Male	1	1	1
Female	0.91 (0.71-1.18)	0.87 (0.68-1.13)	1.05 (0.79-1.39)
Chief/senior physicians	1	1	1
Residents	0.91 (0.64-1.30)	1.09 (0.76-1.56)	1.39 (0.93-2.08)
Job experience > 9 years	1	1	1
Job experience ≤ 9 years	**1.85 (1.35-2.55)**	**1.65 (1.21-2.26)**	1.05 (0.74-1.49)
Cox and Snell Pseudo-R^2^	0.054	0.035	0.012
Nagelkerke's Pseudo-R^2^	0.074	0.047	0.018
Significance level (Chi^2^)	0.000	0.000	0.015

**Demand-control model**			
No strain (low demands/high control)	1	1	
Active job (high demands/high control)	**2.84 (1.99-4.06)**	**1.59 (1.10-2.29)**	1.28 (0.86-1.91)
Passive job (low demands/low control)	**1.80 (1.27-2.54)**	**2.16 (1.53-3.03)**	1.33 (0.92-1.92)
Job strain (high demands/low control)	**3.21 (2.23-4.61)**	**3.08 (2.14-4.42)**	1.04 (0.70-1.57)
**Control variables**			
Male	1	1	1
Female	0.82 (0.64-1.05)	0.78 (0.61-1.00)	1.07 (0.81-1.41)
Chief/senior physicians	1	1	1
Residents	1.00 (0.70-1.42)	0.95 (0.67-1.36)	1.36 (0.91-2.03)
Job experience > 9 years	1	1	1
Job experience ≤ 9 years	**1.53 (1.13-2.08)**	**1.36 (1.01-1.85)**	1.17 (0.84-1.64)
Cox and Snell Pseudo-R^2^	0.059	0.045	0.011
Nagelkerke's Pseudo-R^2^	0.080	0.062	0.017
Significance level (Chi^2^)	0.000	0.000	0.022

^a^Significant odds ratios are bold

All analyses were additionally conducted for men and women separately (not shown in the Tables). Results do not indicate a gender-specific pattern in the association of job stress with quality of care. Beyond that, we also analysed the data stratified by occupational position (subdivided into chief/senior physicians and residents with or without further education; not shown in the Tables). Results are also inconsistent and do not indicate a specific pattern.

## Discussion

In this article psychosocial stress at work and self-reported quality of health care in a nationwide sample of 1,311 clinicians in surgery in Germany were examined. First, the results will be summarised and further analyses of subgroups will be discussed. Then, possible implications and interventions will be considered, followed by a critical discussion of the methodological limitations of our study.

In our study we applied two job stress models (ERI and job strain), and multiple instruments covering different aspects of perceived health care quality in a sample of surgeons. Results indicate that surgeons are exposed to higher job stress compared to the general population and various other professions [4,17,36]. Furthermore, we found a significant association between psychosocial stress at work and care quality in our study. Previous studies that identified an association between job stress and suboptimal quality of care support our results [23,24,26]. However, our findings indicate that the strength of the association varies with the quality and stress indicator. According to the CQS, ERI is only significantly associated with lower psychosocial care. Job strain reveals significant associations with less care quality in terms of all three indicators. A passive job (low demand/low control) is also strongly associated with a lower quality of care respecting the CQS. The frequency of medical errors is marginally associated with both indicators of job stress. In terms of service quality (organisation of care and patient orientation) according to the SERVQUAL, both ERI and job strain are associated with suboptimal service quality.

Our findings imply that interventions aimed at reducing psychosocial stress at work among clinicians may improve the quality of health care [1-3,26]. Promotion of autonomy, provision of adequate support services and a cooperative work environment, as well as promotion of work-life balance are organisations' options in this regard [1,2,37]. At the same time, working overtime, inadequate rewards, high perceived demands and inefficiency at work should be reduced [1,3,37]. Increasing decision latitude, new models of reward and a reorganisation of demands at the workplace may improve both, physicians' health and the quality of care.

To this aim, it is important to improve the doctor's working conditions at different levels [37,38]. At the individual level, the reduction of stressful workplace experiences like overcommitment by stress prevention programs or other stress management interventions could be an option to increase personal well-being at the workplace and to encourage coping skills [2,38]. Studies suggest that social support at work, satisfying work relationships and organisational trust are able to reduce symptoms of work stress and error frequency [2,37,39]. Further possible interventions at the interpersonal level could address improvement of leadership or provision of esteem reward [38] as well as supervisory capacity [3,37]. A reduction of formal hierarchies could promote social support as well. At the structural level, innovations in work organisation, compensatory wage systems or models of gain sharing could be implemented [38]. Interventions on psychosocial work factors (psychological demands, decision latitude, social support, and effort-reward-imbalance) considering these different levels were found to be effective in preventing mental health problems and improving working conditions in a hospital setting [40,41].

Several methodological limitations of our study need to be discussed. This is a cross-sectional study and therefore, no causal inference can be drawn concerning the association between psychosocial work stress and quality of care. A prospective study design with subjective and objective quality indicators is required to avoid this methodological problem. In terms of the quality of the sample, different response rates can be calculated since data collection was based on a stratified probability sample (see Methods). 53% of the hospitals that were contacted took part in the survey and 65% of the clinicians in the participating hospitals responded. Non-response is particularly troublesome if it is associated with working conditions or quality of care in the hospitals. Further analyses published elsewhere [4] indicate that psychosocial stress at work is slightly more pronounced in larger hospitals. As response rates are somewhat lower in larger hospitals, adverse working conditions might be underestimated in our study. To address this problem, the data was weighted for hospital size, ward and occupational position of the surgeons. Although sampling and weighting procedure aimed at generalisability of the results, non-response must be considered as a limiting factor in interpreting our findings.

In terms of the instruments used, psychosocial stress at work was measured by the ERI questionnaire and the JCQ. Both have been tested successfully for their validity [15,27] and were used in previous studies with physicians [5-8,26]. In terms of care quality regarding the physicians' self-assessed performance, we used an instrument called Chirurgisches Qualitätssiegel [28,29] that was derived from the Canadian Physician Achievement Review [30,31]. Although this instrument was specifically developed for the assessment of surgical care by the German Society of Surgery (and the psychometric properties in our study are satisfactory), it cannot be regarded as sufficiently validated. This also holds true for the two questions about the frequency of medical errors and the SERVQUAL instrument [31,32] for assessing the service quality. Although the latter instrument has been used in previous sudies among health care staff [24,34,35] and its psychometric properties are also satisfactory [32-34] the validity of the version used in our study is not yet sufficiently tested. This study is also subject to the problem of common method variance as all variables are based on clinicians' own reports. As associations between psychosocial work and self-reported data might be biased by participants' systematic positive or negative response tendencies [42,43] we adjusted for negative affectivity in additional analyses (not shown in the Tables). The associations between job stress and the perceived quality of care remained significant and the odds ratios were only slightly reduced. Results of these analyses are not shown in detail as the meaning and validity of negative affectivity is ambiguous [27,44,45]. Nevertheless, it is one task for future research to confirm our findings by using a longitudinal design and objective measures of quality of care.

## Conclusions

Despite these limitations, this study shows that theoretical models of psychosocial stress at work can enrich the analysis of effects of working conditions on health care quality. Results indicate that high levels of job stress among clinicians can be a risk to physicians' and patients' health. Furthermore, integrating established models to study psychosocial stress enables theory-driven interventions to prevent stress and to improve working conditions in hospitals.

## Appendix

Measurement of perceived quality of health care according to the "Chirurgisches Qualitätssiegel" (CQS) [28,29]/Canadian Physician Achievement Review (PAR) [30,31]:

"In the following various aspects of physicians' behavior are described. Please rate the quality of your own performance concerning these aspects."

1. Perform surgeries.

2. Assess diagnostic information.

3. Make correct diagnoses.

4. Select appropriate treatments.

5. Maintain medical records.

6. Inform patients about rationale for treatment.

7. Consider psychosocial aspects of illness.

8. Manage health care resources efficiently.

9. Evaluate medical literature to optimize clinical decision making.

10. Participate in implementation of quality improvement programs.

11. Show empathy for patients and their relatives.

12. Involve patients in decision-making.

13. Consider advance health care directives.

Subscales:

Psychosocial care (items 6, 7, 11-13)

Diagnosis/therapy (items 1-4)

Quality assurance (items 5, 8-10)

Scale: "very good", "good", "fair", "not so good", "bad" (+"not applicable")

Measurement of perceived quality of health care according to the "SERVQUAL" [32,33]:

"Declare to what extend do you agree to the following statements concerning your ward and the care at your ward."

1. The ward has up-to-date equipment

2. The ward provides its services at the time they promise to do so.

3. The ward performs the service right the first time.

4. The ward insists on error-free records.

5. The employees of the ward tell the patients exactly when services will be performed.

6. The employees of the ward are always willing to help the patients.

7. The employees of the ward are never too busy to respond your requests.

8. The behaviour of the employees of the ward instils confidence.

9. The employees of the ward are consistently courteous with the patients.

10. The employees of the ward have the knowledge to answer the questions of the patients.

11. The employees of the ward give the patients individual attention.

12. The employees of the ward have patients' best interest at heart.

Subscales:

Organisation of care (items 1-4)

Patient orientation (items 5-12)

Scale: "totally agree", "slightly agree", "slightly disagree", "totally disagree"

## Competing interests

The authors declare that they have no competing interests.

## Authors' contributions

JK drafted the manuscript, analysed and interpreted the data. KGF assisted in data analysing and interpretation. KB was responsible for the sampling procedure and data collection. OvdK conceived and led the project. Moreover, KGF, KB and OvdK contributed to writing and provided feedback on drafts. All authors read and approved the final manuscript.

## Pre-publication history

The pre-publication history for this paper can be accessed here:

http://www.biomedcentral.com/1472-6963/11/109/prepub
